# A life course approach to population oral health

**DOI:** 10.1038/s41415-026-9802-8

**Published:** 2026-11-13

**Authors:** Anja Heilmann, Richard D. Holmes

**Affiliations:** 414161000548653399764 Department of Epidemiology and Public Health, University College London, London, United Kingdom; 500279033376630520697 School of Dental Sciences, Newcastle University, Newcastle, United Kingdom

## Abstract

Oral health inequalities are caused by social, commercial, and political determinants, and remain a persistent national and global challenge. This paper presents an overview of the social determinants of health and highlights the importance of a life course perspective to examine and understand how health inequalities are produced and perpetuated. We discuss how life course theory is linked to wider public health strategies such as the Common Risk Factor Approach and population (or upstream) approaches to prevention, and how it applies to oral health promotion. We emphasise the relevance of a life course approach for dental professionals and policymakers concerned with reducing oral health inequalities and propose how such an approach can be translated into evidence-based oral health policy and practice.

## Key points

- Oral health inequalities present a persistent challenge requiring policies that tackle their social, commercial, and political determinants.
- A life course perspective helps to understand how oral health inequalities are produced and perpetuated throughout life, and to identify strategies and intervention points to address them.
- Dental professionals play an important role in supporting oral disease prevention and advocating for equitable policies to improve population oral health across the lifespan.

## Introduction

Oral diseases constitute a major global public health problem, affecting an estimated 3.7 billion people worldwide.^1^^,^^2^ They are expensive to treat and place a substantial economic burden upon individuals, health services, and wider society, despite being largely preventable.^3^^,^^4^ Dental caries and periodontal disease are the most prevalent oral diseases nationally and globally, and alongside tooth loss have a significant impact on quality of life and daily living, causing pain and functional limitations that affect people's ability to eat, speak, and socialise.^2^ In addition to being highly prevalent, oral diseases are subject to stark inequalities, disproportionately affecting those who are socially and economically disadvantaged.^5^ Fundamentally, these inequalities are not unique to oral health but are seen for most non-communicable diseases as they share the same underlying causes or common risk factors and their social determinants.^6^

In the UK, large oral epidemiological studies have recorded sizable improvements in overall oral health over recent decades.^5^^,^^7^^,^^8^ However, significant concerns remain regarding the size and persistence of oral health inequalities within the population.^5^^,^^9^ Despite recognition that oral diseases and inequalities are driven by social, economic and political factors, oral health interventions too often focus narrowly on downstream approaches such as dental health education and individual behaviour change in what has been termed ‘lifestyle drift', or the shifting of responsibility for health improvement onto individuals.^10^ However, individuals' agency is constrained by the environment and circumstances they live in. On their own, such interventions therefore tend to produce limited and short-term effects, and might even increase inequalities.^11^^,^^12^

Social determinants of health frameworks consider how health and health inequalities are influenced by social contexts.^13^ Importantly, people's experiences unfold over time – health status at any given age is the result not only of current circumstances but also of prior living conditions and experiences, from conception onwards. For example, disadvantage experienced in childhood is consistently linked with adult disease. A life course perspective explicitly considers how social factors influence physical and mental health, or how ‘life gets under the skin'. It seeks to understand how health and disease develop over the lifespan, and how health inequalities are produced and perpetuated over time.^14^

In this article, we present an overview of the social determinants of health, followed by an introduction to life course epidemiology and how it applies to oral health and oral health inequalities. We emphasise the importance of integrating a life course perspective within broader public health strategies aimed at improving oral health and use the example of dental caries to outline intervention and policy options across different life stages.

## Oral health inequalities and the social determinants of health

Health inequalities have been defined as ‘systematic differences in health between different socioeconomic groups in society' that are socially produced and therefore considered avoidable and unjust.^15^ These inequalities are not confined to differences between the most and least advantaged groups. There is a gradient all the way up the social scale, rather than a threshold between the poor and the rest of society above which there are no social differences. Each successively more advantaged group has longer life expectancy and better health, producing the stepwise pattern depicted in epidemiological charts known as the ‘social gradient in health'.^16^ In addition to socio-economic health inequalities, other important dimensions of inequalities include ethnicity, gender, age, geographical area, and disability, as well as inequalities affecting vulnerable and socially excluded groups such as people experiencing homelessness, asylum seekers and refugees. These dimensions often intersect, resulting in the accumulation of disadvantage from discrimination experienced by those who belong to multiple groups.^17^^,^^18^

**Fig. 1:**
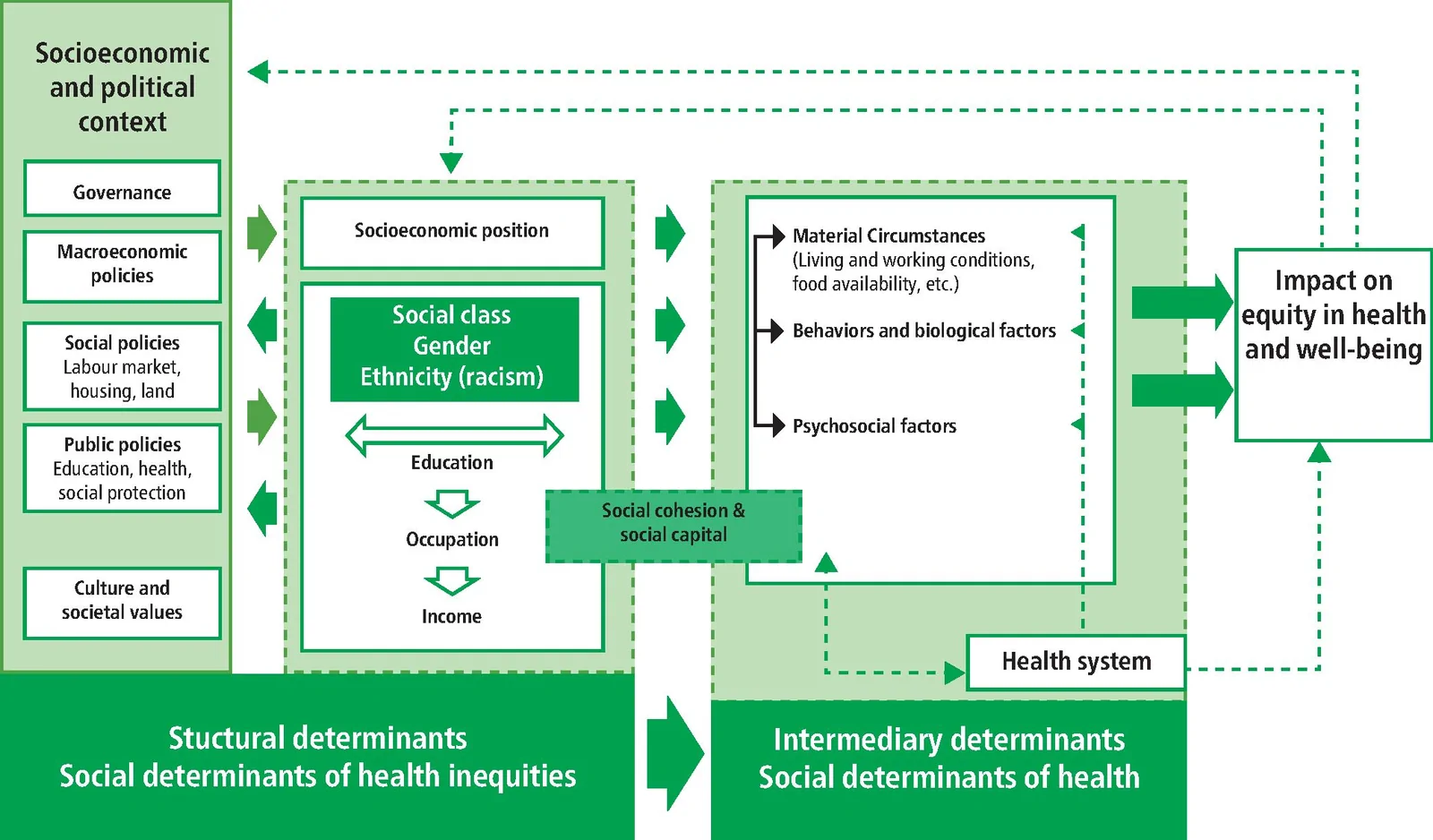
Conceptual Framework for Action on the Social Determinants of Health. Adapted from World Health Organization, 2026^13^

The World Health Organization Commission on Social Determinants of Health produced a framework depicting how the social determinants of health relate to each other and generate inequalities in population health (Fig. 1).^13^ The framework defines social determinants of health as the interrelated conditions in which people are born, grow, live, work, and age. Structural determinants are the socio-political processes generating social hierarchies, which in turn shape intermediary factors such as material resources, psychosocial factors and also behaviours such as diet, smoking and alcohol intake. The unequal distribution of these structural and intermediary factors results in the differential exposure and vulnerability to conditions detrimental to health that give rise to health inequalities across the life course.^13^

Oral conditions such as dental caries and tooth loss are subject to stark socioeconomic inequalities and follow clear social gradients across all stages of life.^9^ For example, the prevalence of dentinal caries in five-year-old children living in the most deprived areas of England is almost three times that of children living in the least deprived areas (35.1% versus 13.5%).^19^ Social gradients in oral health are also consistently seen among adults and in later life, with those who are more disadvantaged being more likely to report tooth loss and poorer oral health-related quality of life.^20^^,^^21^ The persistence of oral health inequalities suggests that their causes go far beyond an individual's ability to access dental services. Furthermore, health-related behaviours only partly explain these patterns.^22^

## A life course approach to oral health

**Fig. 2:**
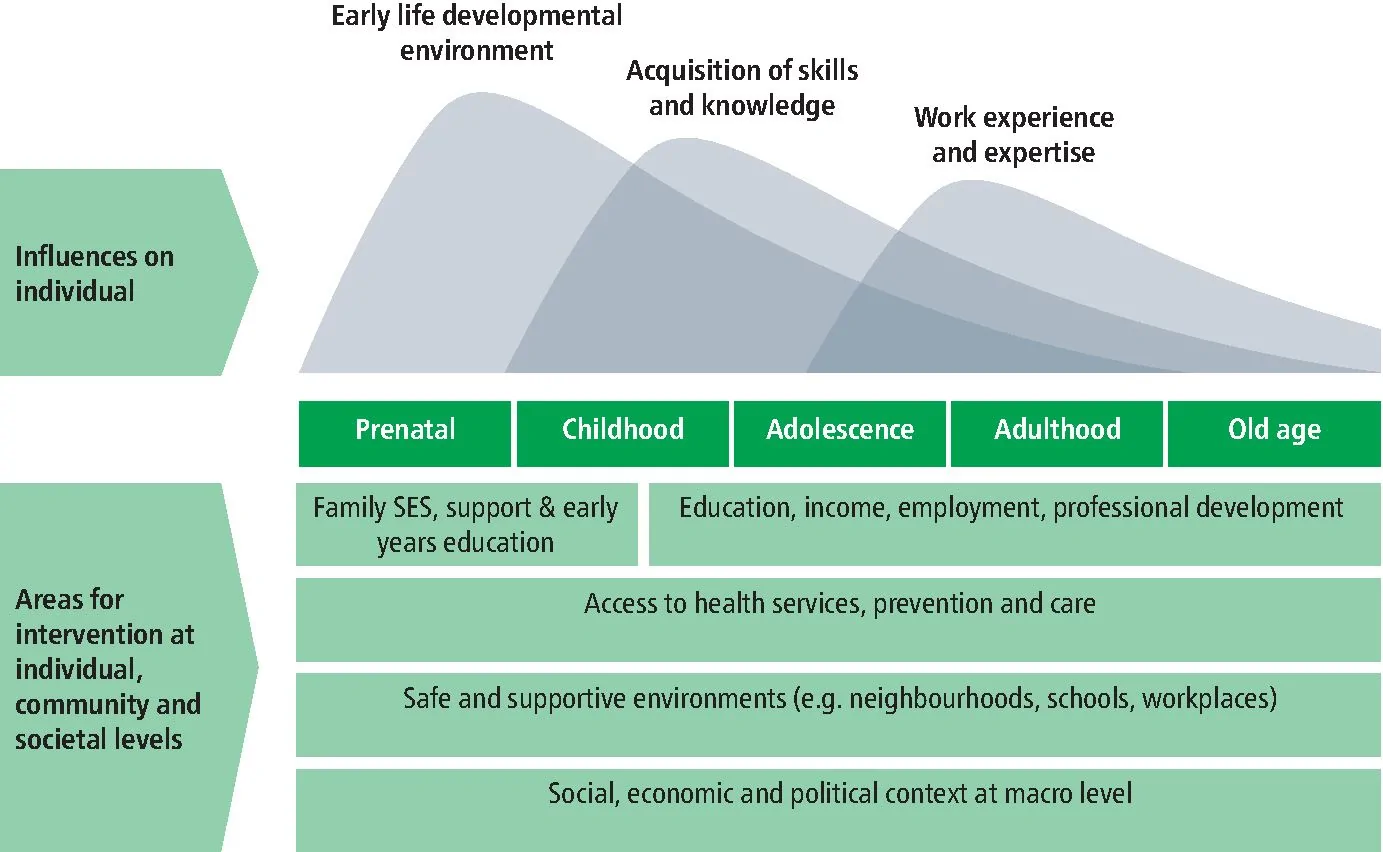
Life course influences on general and oral health. Adapted from Davies, 2012^54^ (SES = socioeconomic status)

Life course epidemiology aims to understand the processes that are causally related to health and disease across the entire lifespan. It is a perspective based on the insights provided by longitudinal studies that follow the same people over long periods of time. Because health inequalities are a result of inequalities in the accumulation of material, social, psychological, and biological advantages and disadvantages over time and across different life stages (Fig. 2), a life course perspective is key to their understanding.^23^^,^^24^ Key life course ‘themes' which help to map a person's journey across the life course have been described in terms of ‘starting well' (from birth), ‘keeping well' (school years), ‘maintaining well' (throughout adult life) through to ‘sustaining well' (into older age).^25^

Similar to other major non-communicable diseases, the most common oral conditions such as dental caries, periodontal disease, and tooth loss are highly prevalent, chronic, and cumulative. Their complex aetiologies include social and environmental factors, meaning they are best understood through a life course approach.^26^ This approach is particularly relevant when attempting to explain how factors in the early years and mid-life may influence oral health in older age.^27^^,^^28^ From a public health perspective, a life course approach contributes to our understanding of oral disease trajectories and inequalities, and can help to inform strategies to address them.^18^^,^^23^

## Life course models and their application to oral health

Life course theoretical models have been developed to explain how life course processes causally influence disease risk. The two main principles are critical/sensitive periods and accumulation of risk over time including pathways. These principles are not mutually exclusive, but rather play out in combination.^14^^,^^29^

### Critical and sensitive periods

Critical and sensitive period models focus on the importance of timing. A critical period is defined as a specific window of time when an exposure can have a causal and irreversible effect, while outside this time window there is no excess risk.^29^ In relation to oral health, critical periods include periods of tooth development, with dental fluorosis a well-known example – exposure to high levels of fluoride can result in dental fluorosis only during amelogenesis (the development of enamel). For the aesthetically important anterior permanent teeth, the critical window for enamel fluorosis spans the first five years of life, with the upper incisors most susceptible during the first three years.^30^

Sensitive periods are a variation of the critical period principle and can be thought of as periods or life stages when an exposure can have a stronger effect than it would have at other times, but where the effect is still modifiable or reversible later on.^31^ Childhood, a period when many habits and food preferences are formed, has been identified as a sensitive period for later life oral health in several studies. In a large UK study following the same children over time to observe caries development from childhood into adolescence, caries levels in permanent teeth were more than four times higher among children who had caries in their primary teeth than among those who did not.^32^ Data from the longitudinal Dunedin Multidisciplinary Health and Development Study (a birth cohort study following the lives of participants born in the early 1970s in Dunedin, New Zealand) demonstrated higher levels of dental caries and periodontal disease among those who experienced socioeconomic adversity in childhood, irrespective of their adult socioeconomic position.^33^

### Accumulation of risk and pathways

Accumulation of risk and pathway models consider how exposures to adverse living conditions or health behaviours accumulate over time and affect health in the form of a dose-response relationship.^34^ Indeed, research consistently shows that longer periods of life spent in socio-economic disadvantage are linked to poorer oral health outcomes.^35^^,^^36^^,^^37^

Risk accumulation also applies to the combination or clustering of different risks, with clustering often resulting from an underlying common cause. For example, the synergistic effect of alcohol and tobacco misuse on oral cancer risk is far greater than the effect of each factor alone.^38^ However, both alcohol and tobacco consumption are influenced by the wider social determinants of health including poorer socio-economic position.^39^ Another example of risk accumulation is exposure to adverse childhood experiences (ACEs), with UK research showing the higher the number of ACEs experienced before the age of 18 years (such as exposure to different forms of abuse, domestic violence, or parental substance misuse), the higher the risk of poor dental health in later life.^40^

Pathway models describe a form of accumulation that considers the sequence of different exposures and how one factor may lead to another, setting in motion a chain of risk. Longitudinal studies from Sweden, China, and England found that childhood socioeconomic disadvantage increased the risk of later life tooth loss through pathways involving poorer socioeconomic position and detrimental health behaviours in adulthood, highlighting the fundamental importance of a good start in life.^35^^,^^41^^,^^42^ Pathways may also play out intergenerationally with disease risk transmitted from one generation to the next, most likely due to a combination of shared social, environmental and behavioural risks as suggested by data from the Dunedin study.^43^ However, socioeconomic circumstances can change across the life course. People can move up or down the social ladder – a concept known as social mobility. Research consistently demonstrates better oral health not only among those who enjoy a stable high socioeconomic position throughout life, but also among those able to move upward, compared to those for whom childhood disadvantage persists through to adulthood.^35^^,^^37^

## Integrating a life course perspective within wider public health strategies

Tackling oral diseases and oral health inequalities requires the integration of a life course perspective with established public health concepts such as the Common Risk Factor Approach (CRFA) and a population (or upstream) approach to prevention.^12^ The CRFA is based on the recognition that most oral and general diseases share common causes such as diet, smoking, and alcohol, as well as their underlying social determinants.^6^ Smoking causes not only periodontal disease and oral cancer, but also a wide range of other noncommunicable diseases. A poor diet that is high in sugar causes not only dental caries, but also obesity, diabetes and cardiovascular disease. It is therefore more efficient, holistic and sustainable to focus on these common causes rather than on single conditions in isolation. The implication of the CRFA is the pooling of efforts and resources through multisectoral collaboration, in other words, the integration of oral and general health perspectives in recommendations, guidelines and prevention programmes. Importantly, behavioural factors do not occur in a vacuum – they are influenced by the social and commercial determinants of health, or the ‘causes of the causes', which must be addressed by creating supportive environments and conditions that empower people to take control over their health.^6^^,^^44^

Prevailing biomedical approaches are mainly based on high-risk strategies targeting only those with the greatest risk of disease. However, for most chronic diseases including dental caries, the majority of new cases occur in those who do not belong to the high-risk group, because they are higher in number,^45^ given that ‘high-risk group' usually defines only the extreme end of the disease distribution. In contrast, population strategies target the underlying social, economic, commercial, and political determinants of health – the root causes of disease – for the population as a whole, shifting the entire population in a favourable direction.^46^ While high-risk strategies often fail to eliminate the underlying causes of disease and require effective and potentially costly screening, population strategies have the potential to prevent far more new cases as exemplified by national and global approaches to tobacco control.^47^ A third option is the directed or targeted population approach, which focuses on population groups with elevated disease risk such as those living in more deprived areas, and do not require individuals to undergo screening. All three strategies have their place and should be sensibly combined.

Population approaches to prevention are often described as upstream interventions, based on the upstream-downstream metaphor,^48^ which uses the image of a river to convey the idea that addressing the root causes of disease, the factors that make people ill in the first place, is far more effective than only providing treatment when disease has already occurred. Upstream or population approaches to improve oral health include policies to create fairer societies and legislation to regulate the manufacturers of harmful products, such as restrictions on lobbying, advertising and marketing, as well as the taxation of sugar, tobacco and alcohol. Midstream interventions are directed locally to create healthy communities and supportive environments in settings such as schools and workplaces, while downstream interventions are about strengthening personal skills and providing preventive services at the dental practice level.^49^

From a life course perspective, strategies are needed that address the social determinants of health at all levels from upstream to downstream and from conception to older age, ensuring supportive conditions throughout life, including during important transitions. To flatten the social gradient, preventive measures should be applied across the whole population in proportion to the level of need, with more resources provided to groups with poorer health – a principle known as proportionate universalism.^44^

**Fig. 3:**
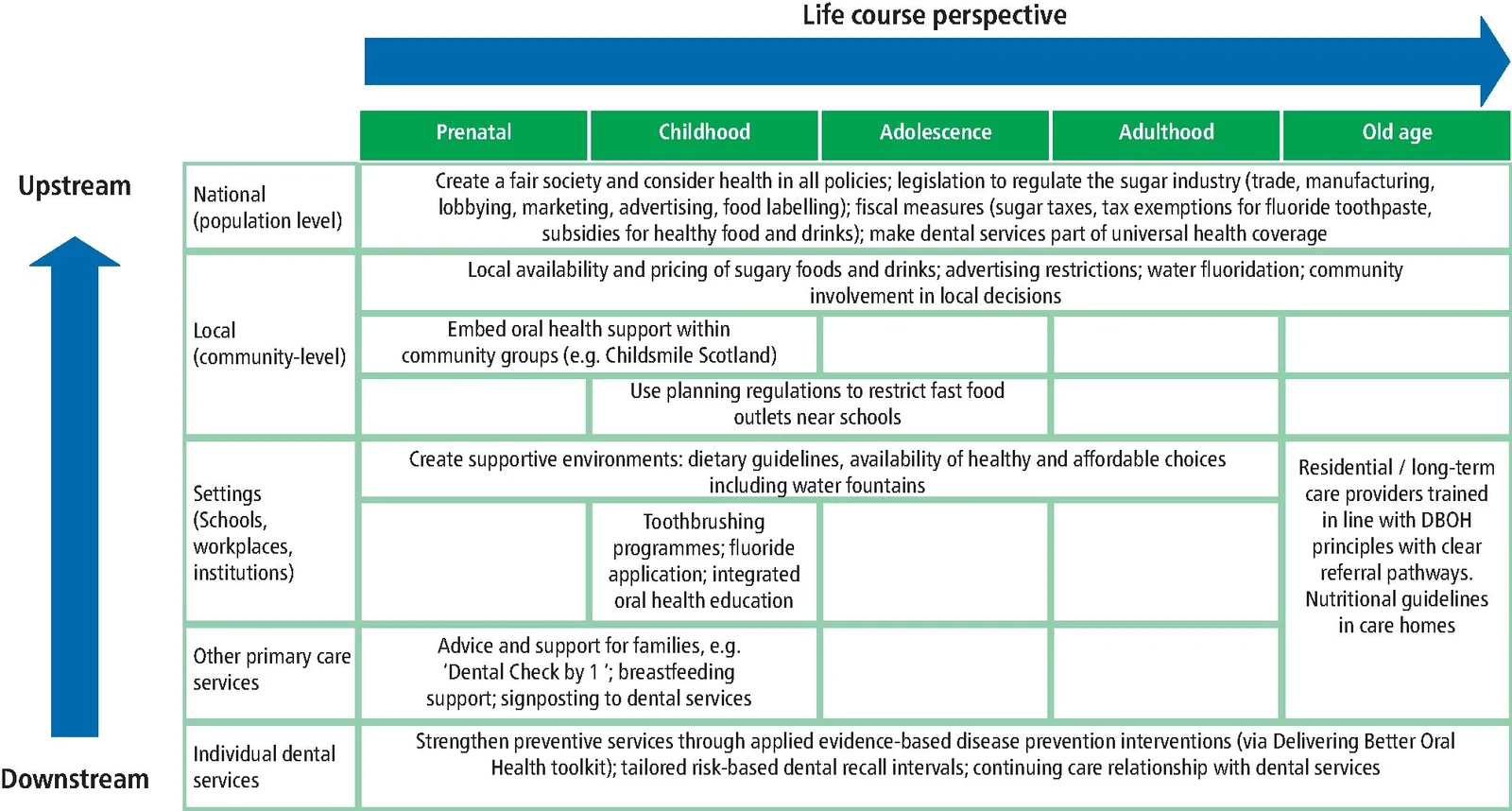
Selected (non-exhaustive) options for dental caries prevention across the life course from an eco-social perspective. Adapted from Wagner *et al.* 2024^34^

Figure 3 uses the example of dental caries to present a range of interventions that can be applied at different life stages, from upstream to downstream approaches including the evidence-based recommendations for prevention outlined in the Delivering Better Oral Health (DBOH) toolkit.^50^

## Recommendations for policy and practice

As we have outlined, there are real limitations with health care professionals and policy makers focusing solely upon high-risk individuals and behaviour change interventions at the expense of approaches that address the underlying social determinants of health.^12^ A life course approach emphasises the need for multi-sectoral action and interventions addressing the structural drivers of inequality. A sensible combination of downstream, midstream, and upstream approaches supporting good oral health across the entire life course is needed if oral diseases and oral health inequalities are to be effectively and sustainably reduced.^2^^,^^51^ Such a life course perspective can help policymakers to identify opportunities for intervention at different levels and life stages. While early intervention is critical, prevention strategies must consider the needs of all age groups and include support for adults and older people, whose oral health needs are becoming more complex in an ageing population retaining their teeth for much longer than in the past.^28^

For dental teams, adopting a life course perspective means meeting patients with compassion and without judgement. Through continuity of care, dental professionals can build trust and lifelong positive relationships with their patients, with regular appointments not only linked to better oral health over time,^52^ but also presenting valuable opportunities for prevention. Evidence-based toolkits based on proportionate universalism such as DBOH provide a highly accessible resource to inform prevention-based activity to help improve patients' oral and general health across the life course, such as motivational interviewing and brief advice on oral hygiene, diet, smoking, and alcohol.^50^ Where appropriate, dental professionals can refer their patients to preventive services delivered in primary care, as well as signpost non-clinical local services offering support with broader needs (social prescribing). While much of the content of DBOH is typically applied downstream, dental teams can promote oral health equity in multiple ways, for example by ensuring that practices are accessible for disabled people and vulnerable population groups, and through working with their local communities on outreach programmes for schools and care homes. The dental community also has a key role in advocating for midstream and upstream policies conducive to oral health, working across sectors with local authorities and national public health bodies to lobby e.g., for the regulation of harmful products, dietary guidelines in institutions, and other measures outlined in Figure 3.^53^

To continue meeting the oral health needs and expectations of a population undergoing rapid demographical change, stakeholders including policy makers, workforce providers and dental teams need to consider how to support patients long-term across the life course, as opposed to the focus often being upon single, cross-sectional courses of care or treatment ‘activity'.^25^ However, it is acknowledged there are constraints with existing models of care that would need addressing in order to optimise this approach. Further embracing skill mix and the diversity of the oral health workforce together with greater integration of the profession with other health and social care professionals may help to facilitate the provision of care and support to patients across different stages of their lives.

## Conclusion

Oral health is a dynamic process rooted in the conditions and environments experienced throughout life. A life course approach considers how cumulative exposures throughout life shape oral health trajectories and inequalities and directs attention to their underlying social, economic, and environmental determinants. Dental teams have a central role to play in oral disease prevention and advocating for the promotion of greater oral health equity across the entire lifespan.
